# Estimating the Number of Latent Ranks of the Fugl-Meyer Assessment Score for the Affected Upper Extremity After Stroke

**DOI:** 10.7759/cureus.84210

**Published:** 2025-05-16

**Authors:** Kensuke Hara, Yuta Tauchi, Keisuke Hanada, Takashi Takebayashi

**Affiliations:** 1 Graduate School of Rehabilitation, Osaka Metropolitan University, Osaka, JPN; 2 Department of Rehabilitation Medicine, Hyogo Medical University Sasayama Medical Center, Tamba-Sasayama, JPN; 3 Faculty of Rehabilitation, Shijonawate Gakuen University, Osaka, JPN

**Keywords:** fugl-meyer assessment-upper extremity, latent rank theory, rehabilitation, stroke, upper extremity

## Abstract

Many clinical stroke rehabilitation studies have adopted the upper extremity motor section of the Fugl-Meyer Assessment (FMA-UE). In addition, some clinical studies use specific FMA-UE scores as inclusion criteria. However, it remains unclear whether it is appropriate to determine the criterion based on the total score of FMA-UE. This study aimed to determine a highly valid criterion using the latent rank theory (LRT) that can estimate the number of latent ranks of FMA-UE. This was a multicenter cross-sectional study; patients with stroke were recruited from 25 hospitals between March 2018 and April 2022. For all patients, FMA-UE results and participant information were collected. The collected FMA-UE data were divided into proximal and distal items and verified the dimensionality of the data. After that, the LRT was used to determine the latent ranks. Seven ranks were considered the most appropriate for proximal and distal items when estimating the number of latent ranks. These results suggest that FMA-UE has high construct validity. Furthermore, we recommend the novel interpretability of FMA-UE, which previous studies have yet to find. Although this cross-sectional study cannot directly guide stroke patients' recovery processes, it may be practical for optimizing the difficulty of stroke rehabilitation.

## Introduction

The Fugl-Meyer Assessment (FMA) is widely used as a functional assessment for stroke patients [1]. The upper extremity motor section of the Fugl-Meyer Assessment (FMA-UE) is endorsed by the guidelines of the American Heart Association and the American Stroke Association as a valid evaluation scale for evaluating rehabilitation care during the recovery phase of adult stroke patients [2]. The FMA-UE is known for its reliability and validity, making it an essential outcome measure for intervention planning based on the severity of stroke and for evaluating the effectiveness of upper extremity rehabilitation [3]. However, there are two further concerns regarding the responsiveness and interpretability of FMA-UE: One relates to the cutoff values, and the other refers to total scoring.

First, the cutoff values based on the minimal clinically important difference (MCID) are not precise responsiveness because each subitem has different levels of difficulty, and the weight of each score on a three-point ordinal scale is theoretically uneven [4,5]. The movement of "shoulder flexion to 90°" differs in difficulty from "forearm pronation/supination, elbow at 90°." Also, within the same item, the difference observed between scores of 0-1 and 1-2 is not equivalent. Therefore, the item response theory has recently been applied to the scale analysis of the FMA-UE [6,7]. These studies investigated the interpretability of the FMA-UE by estimating the discrimination and difficulty of all subitems and determining the test response function of the total score [6,7].

One of the previous studies using Rasch analysis was used to sort the FMA-UE items to estimate cutoff values for the difficulty of each item and the severity classification of the paretic extremity based on logit values calculated from the total score [7]. These findings may be useful for fine-tuning individual patient programs [6,7]. However, the risk of false positives and false negatives remains for cutoff values because Rasch analysis presumes the "continuity" of test items, leaving a risk of false positives and negatives even if cutoffs are based on logit values. Again, the responsiveness and interpretability of FMA-UE remain open to discussion.

These concerns may be solved using the latent rank theory (LRT) [8-10]. LRT assumes the "ordinality" of test items. LRT is a method developed by Shojima in 2007, who proposed a general framework for ordinal data analysis based on neural networks [8-10]. LRT encompasses the neural test theory [9] and utilizes self-organizing maps [11] and generative topographic mapping [12]. Several previous studies have utilized LRT [13,14]. Since LRT does not impose mathematical assumptions beyond the ordinality of latent variables, it can flexibly analyze various data and richly represent models. As a result, interpretations are easier than with Rasch analysis. The previous study using LRT for Berg Balance Scale (BBS) has also allowed for explaining differences in treatment efficacy according to subject severity and facilitating the development of treatments for patients with specific severity [15]. The use of LRT for FMA-UE means that feedback to therapists and subjects is clearer and easier because it assumes "ordinality," whereas Rasch analysis, which assumes "continuity," requires an understanding of the scale due to the detailed item difficulty. Therefore, new interpretations using LRT for FMA-UE are needed for interpretations that complement previous studies to date.

Second, the interpretation based on total scores of FMA-UE may not be valid from a neuroanatomical or psychometric perspective. The previous study reported that 30 items of FMA-UE, excluding reflex activities and normal reflexes, can be treated as unidimensional [16]. The unidimensionality could be interpreted as a given FMA-UE score to estimate severity or to assess based on the MCID. Nevertheless, the total score does not tell us whether the paresis is proximally or distally dominant. Previous studies have shown that the lesion site in stroke may lead to dominance in proximal or distal paralysis and that recovery does not necessarily follow a proximal-to-distal gradient [17]. There are also studies using Rasch analysis for the wrist and finger items alone in previous FMA-UE research [18]. Thus, it would be more meaningful to estimate the latent ranks of FMA-UE separately for proximal and distal sections.

This study estimates latent ranks for the proximal and distal items of FMA-UE and clarifies the relationship between each rank and FMA-UE subitems. If the latent ranks for proximal and distal items in FMA-UE can be explained, new interpretations that complement previous studies may be possible. This would standardize the interpretation of FMA-UE scores in clinical settings, facilitate the identification of treatment targets, and contribute to shared decision-making with patients and multidisciplinary teams.

## Materials and methods

Study design and participants

This cross-sectional study recruited patients from 25 hospitals between March 2018 and April 2022. The selected hospitals were those located in Japan that admit stroke patients and where consent for this study was obtained. The inclusion criteria were as follows: (i) patients with unilateral supratentorial stroke occurring for the first time and (ii) those aged ≥20 years. The exclusion criteria were as follows: (i) patients with significant pain and stiffness in the more affected upper extremity, (ii) those who were currently receiving rehabilitation at the time of the conduction of the study, (iii) those with severe cognitive deficits that would preclude clinical evaluations, and (iv) those with other severe medical conditions.

All participants underwent standard rehabilitation programs, such as physical and occupational therapy. The study was approved by the Committee on Research Ethics of the Graduate School of Comprehensive Rehabilitation of Osaka Prefecture University (approval number: 2021-205) and registered with the University Hospital Medical Information Network (UMIN) Clinical Trials Registry (UMIN000030366). It was conducted according to the tenets of the Declaration of Helsinki, and written informed consent was obtained from all participants.

Instruments

The FMA-UE was used to evaluate upper extremity motor paralysis and to measure the recovery index [1]. The FMA-UE is composed of four subscales and 33 items as follows: (A) "shoulder/elbow/forearm" (18 items), (B) "wrist" (five items), (C) "hand" (seven items), and (D) "coordination/speed" (three items) [1]. Each item is rated on a three-point ordinal scale (0, cannot perform; 1, performs partially; and 2, performs fully), aside from items 1 and 2 (0, none; 2, can be elicited), and the total score ranges from 0 to 66. We used the FMA-UE Japanese version of the training manual [3]. To prevent measurement bias, all the raters were trained to administer the FMA-UE before commencing the study. The modified Rankin Scale (mRS) was used to evaluate the disability and handicap of patients after stroke [19]. The Japanese version of the mRS with an expanded guidance scheme was used [20]. The total mRS score ranged from 0 to 6 points (0, normal; 1, normal to mild; 2, mild; 3, moderate; 4, moderate to severe; and 5, severe).

Data analysis

All gathered data were promptly analyzed after collection. Initially, the data were examined to identify missing values for specific variables or participants. Subsequently, the dimensionality of the data was verified. After that, the latent rank theory (LRT) was used to determine the latent ranks for items in which one-dimensionality was confirmed by checking for dimensionality.

Dimensionality

Dimensionality is an underlying attribute that needs to be measured by the FMA-UE [21]. Furthermore, dimensionality signifies "construct validity," with various aspects falling under the validity framework. One-dimensionality, mandatory in the LRT, represents only one of the latent traits, and the one-factor model represents this via factor analysis. Although FMA-UE comprises reflex and voluntary movement items, it is considered unidimensional in explaining the concept of upper extremity function (33-item model with one-factor structure). We divided FMA-UE into proximal (shoulder, elbow, forearm, and coordination) and distal (wrist and finger) items and examined the dimensionality of each. A polyserial correlation coefficient (PCC) and confirmatory factor analysis (CFA) were conducted separately for proximal and distal items. PCC allows us to confirm the relationship between the measured object and each item in the entire scale, and when this value is high, it can be interpreted as a coherent scale [22]. In this study, the scale was considered unidimensional when the PCC for all items was 0.2 or higher [22]. In addition, CFA was conducted on the FMA-UE to confirm whether the model was unidimensional. We utilized three indices of model fit: the comparative fit index (CFI), Tucker-Lewis index (TLI), and root mean square error of approximation (RMSEA). The adequacy of model fit was defined as a CFI and TLI of ≥0.95 [23]. RMSEA critical values of 0.08-0.10 and <0.08 indicated a mediocre fit and a good fit, respectively [24].

Latent rank theory

The LRT involved estimating the latent ranks for all six distribution specifications (including "no distribution specification," "uniform distribution," and "normal distribution") and applying monotonically increasing constraints (with and without) while examining models with latent ranks ranging from four to eight. The selection criteria for the models were based on the ease of interpretation, information criterion values, and the model fit index. The information criteria were Akaike's information criterion (AIC), Bayesian information criterion (BIC), and consistent AIC (CAIC) determined by comparing each index relative to each other [8-10]. If each value was low, the model was good. The model fit index was RMSEA. Latent ranks were interpreted based on the FMA-UE factor structure and item content, test reference profile (TRP), item reference profile (IRP), and item category reference profile (ICRP) [8-10]. TRP is an expected value that indicates how much the patient, estimated to belong to each latent rank, will score on the total score of FMA-UE. IRP stated the characteristics of each item of FMA-UE by latent rank, and the average score for each latent rank was calculated from the category response rate for each item of FMA-UE. The ICRP calculates the probability of the patient for each of the latent ranks for each item of FMA-UE to be scored 0, 1, or 2 points on the prime score. Based on the objectives of this study and the results of the above profiles, we interpreted the characteristics of the latent ranks of FMA-UE.

Statistical analysis

The results were analyzed using the following software tools: (i) R version 4.1.2 (R Foundation for Statistical Computing, Vienna, Austria) for statistical analysis to confirm the characteristics of the participants, CFA, generalized linear model, and Tukey's multiple comparisons, and (ii) Exametrika version 5.5 (Shojima K, Tokyo, Japan), which is a software program developed based on the neural network theory to analyze PCC and LRT.

## Results

A total of 509 participants were included in the study. There were no participants who were excluded. The characteristics of the participants in the final analysis are presented in Table 1. The participants were mainly patients with subacute-to-chronic stroke. The mean FMA-UE scores were 39.9. The distribution of the percentages of stroke severity was similar to those of previous studies [6,7].

**Table 1:** Patient characteristics (n=509) Values are expressed as numbers (percentages) or as median (interquartile range) mRS, modified Rankin Scale; FMA-UE, upper extremity motor section of the Fugl-Meyer Assessment

Patient characteristics	n=509	
Age (years)	67.8 (59-78)	
Sex		
Male	311 (61.1)	
Days since stroke	133.1 (36.0-120.0)	
Hand		
Right	482 (95)	
Stroke type		
Ischemic	310 (61)	
Hemorrhagic	198 (39)	
Stroke location		
Right	296 (58)	
Left	213 (42)	
FMA-UE		
Total score	39.9 (23.0-58.0)	
Subscale score		
A. Shoulder/elbow/forearm	23.9 (16.0-33.0)	
B. Wrist	5.4 (1-9)	
C. Hand	8.3 (3.0-14.0)	
D. Coordination/speed	2.3 (0-5)	
mRS score		
0	4 (1)	
1	46 (9)	
2	133 (26)	
3	120 (24)	
4	174 (34)	
5	32 (6)	

Dimensionality

In the PCC of the proximal items, the coefficients were more significant than 0.2 for 16 items, except for the two reflex items (Table 2). In the PCC of the distal items, the coefficients were more significant than 0.2 for all items (Table 2). Based on this analysis, we removed these two reflex items from the FMA-UE scale. The one-factor analysis with CFA results showed that the CFI/TLI value was 0.99 for both proximal and distal models. The RMSEA value was 0.096 (90% confidence interval {CI}: 0.090-0.102) for the proximal item model and 0.101 (90% CI: 0.090-0.111) for the distal item model.

**Table 2:** Polyserial correlation coefficient (PCC) for the proximal and distal item Fugl-Meyer Assessment of the upper extremity The scale was judged to be unidimensional when the PCC for all items was 0.2 or higher SD: standard deviation

Proximal task	Item	Mean	SD	PCC
Reflex activity	Reflex biceps	1.929	0.370	0.041
	Reflex triceps	1.780	0.626	0.160
Flexor synergy	Forearm supination	1.320	0.764	0.864
	Elbow flexion	1.619	0.646	0.893
	Shoulder abduction	1.361	0.728	0.870
	Shoulder external rotation	1.255	0.800	0.902
	Scapular elevation	1.444	0.718	0.880
	Scapular retraction	1.358	0.730	0.855
Extensor synergy	Forearm pronation	1.521	0.762	0.898
	Elbow extension	1.511	0.723	0.890
	Shoulder adduction/internal rotation	1.523	0.725	0.922
Movement combining	Hand to lumber spine	1.413	0.803	0.875
	Shoulder flexion to 90°	1.224	0.877	0.930
	Forearm pronation/supination, elbow at 90°	1.244	0.830	0.879
Movement out of synergy	Shoulder abduction 0°-90°	1.147	0.867	0.915
	Shoulder flexion 90°-180°	0.817	0.830	0.911
	Forearm pronation/supination, elbow at 0°	1.004	0.835	0.908
Normal reflex	Normal reflex	0.448	0.791	0.742
Coordination/speed	Tremor	0.843	0.898	0.833
	Dysmetria	0.758	0.859	0.856
	Speed	0.690	0.857	0.902
Distal task	Item	Mean	SD	PCC
Wrist	Wrist stability, elbow at 90°	1.269	0.885	0.910
	Wrist pronation/supination, elbow at 90°	1.169	0.832	0.882
	Wrist stability, elbow at 0°	1.116	0.901	0.912
	Wrist pronation/supination, elbow at 0°	1.024	0.834	0.911
	Wrist circumduction	0.864	0.729	0.894
Hand	Finger mass flexion	1.489	0.700	0.846
	Finger mass extension	1.338	0.789	0.873
	Hook grasp	1.041	0.941	0.848
	Abduct thumb	0.974	0.868	0.863
	Oppose thumb and index finger pads	1.069	0.899	0.903
	Cylindrical grasp	1.141	0.885	0.889
	Spherical grasp	1.238	0.879	0.888

Latent rank theory

For latent ranks in FMA-UE, rank 7 was the most appropriate for proximal and distal items (no monotonically increasing constraints and no distribution specification), considering the ease of interpretation and information criterion values (Table 3 and Appendices). Although rank 8 has a lower value than rank 7 when considering the information criterion value, rank 7 was selected for this study because the distribution of subjects and the interpretation of IRP would be complex. The TRP, IRP, and ICRP values increased as the rank increased (Tables 4, 5, 6).

**Table 3:** Information criterion values of latent ranks for proximal and distal items The selection criteria for the models were based on the ease of interpretation, information criterion values, and the model fit index. The information criteria were AIC, BIC, and CAIC, determined by comparing each index relative to each other, and if each value was low, the model was good. The model fit index was RMSEA, with critical values of RMSEA ranging from 0.08-0.10 to <0.08, indicating mediocre and good fit, respectively AIC, Akaike's information criterion; BIC, Bayesian information criterion; CAIC, consistent AIC; RMSEA, root mean square error of approximation

Information criterion values of latent ranks for proximal items						Information criterion values of latent ranks for distal items					
Proximal items						Distal items					
Distribution specifications	Rank	No monotonically increasing constraints				Distribution specifications	Rank	No monotonically increasing constraints			
		AIC	CAIC	BIC	RMSEA			AIC	CAIC	BIC	RMSEA
No distribution specification	4	1921.114	-2254.38	-1456.38	0.082	No distribution specification	4	1221.877	-1415.28	-911.277	0.082
	5	1594.941	-2381.72	-1621.72	0.078		5	963.947	-1547.63	-1067.63	0.077
	6	1410.028	-2367.8	-1645.8	0.076		6	821.741	-1564.26	-1108.26	0.074
	7	1321.364	-2257.63	-1573.63	0.076		7	748.417	-1512	-1080	0.073
	8	1260.047	-2120.12	-1474.12	0.076		8	704.062	-1430.78	-1022.78	0.073
Uniform distribution	4	2276.484	-1899.01	-1101.01	0.087	Uniform distribution	4	1548.36	-1088.79	-584.794	0.09
	5	2039.644	-1937.02	-1177.02	0.085		5	1371.085	-1140.49	-660.49	0.087
	6	1841.669	-1936.16	-1214.16	0.084		6	1309.466	-1076.53	-620.53	0.087
	7	1713.821	-1865.17	-1181.17	0.083		7	1198.059	-1062.36	-630.359	0.086
	8	1665.966	-1714.2	-1068.2	0.084		8	1157.521	-977.317	-569.317	0.087
Normal distribution	4	4193.492	17.998	815.998	0.111	Normal distribution	4	2994.533	357.379	861.379	0.117
	5	4112.779	136.119	896.119	0.112		5	2259.851	-251.725	228.275	0.106
	6	2593.239	-1184.59	-462.589	0.095		6	1861.661	-524.335	-68.335	0.1
	7	2588.549	-990.445	-306.445	0.097		7	1581.835	-678.582	-246.582	0.096
	8	2200.833	-1179.33	-533.329	0.093		8	1908.482	-226.357	181.643	0.106
Distribution specifications	Rank	Monotonically increasing constraints				Distribution specifications	Rank	Monotonically increasing constraints			
		AIC	CAIC	BIC	RMSEA			AIC	CAIC	BIC	RMSEA
No distribution specification	4	1921.114	-2254.38	-1456.38	0.082	No distribution specification	4	1221.877	-1415.28	-911.277	0.082
	5	1594.941	-2381.72	-1621.72	0.078		5	963.947	-1547.63	-1067.63	0.077
	6	1410.028	-2367.8	-1645.8	0.076		6	821.741	-1564.26	-1108.26	0.074
	7	1321.364	-2257.63	-1573.63	0.076		7	748.417	-1512	-1080	0.073
	8	1260.047	-2120.12	-1474.12	0.076		8	704.062	-1430.78	-1022.78	0.073
Uniform distribution	4	2276.484	-1899.01	-1101.01	0.087	Uniform distribution	4	1548.36	-1088.79	-584.794	0.09
	5	2039.644	-1937.02	-1177.02	0.085		5	1371.085	-1140.49	-660.49	0.087
	6	1841.669	-1936.16	-1214.16	0.084		6	1309.466	-1076.53	-620.53	0.087
	7	1713.821	-1865.17	-1181.17	0.083		7	1198.059	-1062.36	-630.359	0.086
	8	1665.966	-1714.2	-1068.2	0.084		8	1157.521	-977.317	-569.317	0.087
Normal distribution	4	3635.697	-539.797	258.203	0.105	Normal distribution	4	3034.746	397.592	901.592	0.118
	5	4112.779	136.119	896.119	0.112		5	2026.498	-485.077	-5.077	0.101
	6	2491.022	-1286.81	-564.805	0.094		6	1841.294	-544.702	-88.702	0.1
	7	2588.549	-990.445	-306.445	0.097		7	1581.835	-678.582	-246.582	0.096
	8	2200.833	-1179.33	-533.329	0.093		8	1572.161	-562.678	-154.678	0.098

**Table 4:** Item reference profile (IRP) for proximal and distal items The redder the gradient, the higher the IRP value

Proximal items	Rank 1	Rank 2	Rank 3	Rank 4	Rank 5	Rank 6	Rank 7
Elbow flexion	0.893	1.156	1.539	1.799	1.919	1.973	1.992
Shoulder adduction/internal rotation	0.662	0.948	1.388	1.708	1.877	1.958	1.986
Forearm pronation	0.614	0.934	1.415	1.749	1.903	1.964	1.984
Elbow extension	0.68	0.956	1.372	1.674	1.84	1.934	1.972
Scapular elevation	0.64	0.855	1.216	1.532	1.749	1.888	1.949
Hand to lumber spine	0.482	0.773	1.23	1.576	1.771	1.892	1.951
Shoulder abduction	0.566	0.785	1.121	1.401	1.622	1.802	1.899
Scapular retraction	0.593	0.77	1.079	1.377	1.62	1.799	1.889
Forearm supination	0.5	0.744	1.099	1.37	1.58	1.764	1.865
Shoulder external rotation	0.319	0.564	0.96	1.296	1.559	1.776	1.894
Forearm pronation/supination, elbow at 90°	0.297	0.548	0.945	1.282	1.551	1.775	1.892
Shoulder flexion to 90°	0.187	0.409	0.824	1.247	1.6	1.845	1.947
Shoulder abduction 0°-90°	0.154	0.326	0.687	1.109	1.487	1.762	1.893
Forearm pronation/supination, elbow at 0°	0.12	0.27	0.562	0.9	1.255	1.567	1.722
Tremor	0.069	0.155	0.346	0.623	0.998	1.375	1.573
Shoulder flexion 90°-180°	0.042	0.12	0.313	0.604	0.971	1.339	1.552
Dysmetria	0.049	0.126	0.294	0.539	0.883	1.246	1.445
Speed	0.014	0.051	0.168	0.404	0.775	1.177	1.408
Normal reflex	0.025	0.048	0.098	0.195	0.409	0.736	0.97
Distal items	Rank 1	Rank 2	Rank 3	Rank 4	Rank 5	Rank 6	Rank 7
Finger mass flexion	0.746	0.951	1.298	1.592	1.788	1.911	1.961
Finger mass extension	0.425	0.66	1.066	1.432	1.702	1.877	1.94
Wrist stability, elbow at 90°	0.212	0.475	0.953	1.38	1.686	1.888	1.966
Spherical grasp	0.237	0.477	0.901	1.297	1.606	1.834	1.936
Wrist flexion/extension, elbow at 90°	0.207	0.44	0.844	1.199	1.48	1.729	1.868
Cylindrical grasp	0.171	0.359	0.734	1.132	1.47	1.741	1.875
Wrist stability, elbow at 0°	0.101	0.271	0.635	1.06	1.462	1.775	1.912
Oppose thumb and index finger pads	0.12	0.28	0.608	0.976	1.34	1.677	1.852
Hook grasp	0.156	0.277	0.544	0.887	1.271	1.63	1.822
Wrist flexion/extension, elbow at 0°	0.116	0.276	0.602	0.955	1.285	1.592	1.762
Abduct thumb	0.131	0.244	0.495	0.833	1.204	1.539	1.713
Wrist circumduction	0.131	0.282	0.553	0.819	1.069	1.311	1.45

**Table 5:** Item category reference profile (ICRP) for proximal items The ICRP calculates the probability of the patient for each of the latent ranks for each item of FMA-UE to be scored 0, 1, or 2 points on the prime score FMA-UE: upper extremity motor section of the Fugl-Meyer Assessment

Proximal item	Score	Rank 1	Rank 2	Rank 3	Rank 4	Rank 5	Rank 6	Rank 7
Forearm supination	0	0.578	0.409	0.184	0.059	0.018	0.006	0.002
	1	0.344	0.439	0.532	0.513	0.384	0.225	0.131
	2	0.078	0.152	0.283	0.428	0.598	0.769	0.867
Elbow flexion	0	0.299	0.203	0.082	0.02	0.003	0	0
	1	0.51	0.438	0.296	0.162	0.075	0.026	0.008
	2	0.192	0.359	0.622	0.818	0.922	0.974	0.992
Shoulder abduction	0	0.49	0.339	0.141	0.034	0.005	0.001	0
	1	0.454	0.537	0.598	0.531	0.367	0.197	0.101
	2	0.056	0.124	0.262	0.435	0.627	0.803	0.899
Shoulder external rotation	0	0.708	0.515	0.239	0.073	0.017	0.003	0.001
	1	0.266	0.406	0.561	0.559	0.407	0.218	0.105
	2	0.027	0.079	0.2	0.369	0.576	0.779	0.895
Scapular elevation	0	0.429	0.299	0.129	0.037	0.011	0.004	0.001
	1	0.503	0.548	0.527	0.394	0.229	0.104	0.048
	2	0.069	0.153	0.344	0.569	0.76	0.892	0.951
Scapular retraction	0	0.452	0.333	0.169	0.066	0.023	0.009	0.006
	1	0.503	0.565	0.584	0.49	0.333	0.184	0.099
	2	0.045	0.103	0.247	0.443	0.643	0.807	0.895
Forearm pronation	0	0.53	0.371	0.163	0.047	0.011	0.002	0
	1	0.326	0.324	0.26	0.156	0.075	0.032	0.015
	2	0.144	0.305	0.578	0.796	0.914	0.966	0.985
Elbow extension	0	0.441	0.302	0.126	0.035	0.008	0.002	0
	1	0.437	0.44	0.376	0.257	0.143	0.062	0.027
	2	0.122	0.258	0.498	0.708	0.849	0.936	0.973
Shoulder adduction/internal rotation	0	0.453	0.311	0.128	0.031	0.005	0.001	0
	1	0.432	0.429	0.356	0.23	0.113	0.041	0.014
	2	0.115	0.259	0.516	0.739	0.882	0.959	0.986
Hand to lumber spine	0	0.627	0.459	0.218	0.07	0.017	0.003	0.001
	1	0.263	0.309	0.333	0.284	0.195	0.102	0.048
	2	0.109	0.232	0.449	0.646	0.788	0.895	0.951
Shoulder flexion to 90°	0	0.837	0.668	0.39	0.175	0.066	0.019	0.005
	1	0.139	0.255	0.396	0.402	0.269	0.117	0.042
	2	0.024	0.077	0.214	0.423	0.665	0.864	0.952
Forearm pronation/supination, elbow at 90°	0	0.749	0.558	0.285	0.113	0.044	0.017	0.007
	1	0.205	0.336	0.485	0.493	0.36	0.19	0.094
	2	0.046	0.106	0.23	0.395	0.596	0.792	0.899
Shoulder abduction 0°-90°	0	0.859	0.718	0.454	0.206	0.065	0.013	0.003
	1	0.128	0.238	0.406	0.479	0.384	0.211	0.102
	2	0.013	0.044	0.14	0.315	0.552	0.776	0.896
Shoulder flexion 90°-180°	0	0.96	0.889	0.724	0.504	0.283	0.119	0.047
	1	0.037	0.101	0.239	0.389	0.464	0.424	0.355
	2	0.002	0.01	0.037	0.107	0.254	0.458	0.598
Forearm pronation/supination, elbow at 0°	0	0.886	0.749	0.503	0.275	0.124	0.043	0.014
	1	0.108	0.232	0.431	0.549	0.498	0.348	0.249
	2	0.006	0.019	0.065	0.175	0.379	0.609	0.737
Normal reflex	0	0.979	0.962	0.929	0.874	0.758	0.581	0.454
	1	0.018	0.028	0.044	0.058	0.075	0.102	0.121
	2	0.003	0.01	0.027	0.069	0.167	0.317	0.425
Tremor	0	0.959	0.902	0.774	0.595	0.377	0.178	0.079
	1	0.013	0.04	0.105	0.187	0.248	0.268	0.269
	2	0.028	0.058	0.12	0.218	0.375	0.553	0.652
Dysmetria	0	0.969	0.919	0.805	0.639	0.421	0.207	0.099
	1	0.012	0.037	0.097	0.183	0.275	0.339	0.356
	2	0.018	0.045	0.098	0.178	0.304	0.454	0.544
Speed	0	0.988	0.958	0.869	0.71	0.488	0.271	0.156
	1	0.01	0.033	0.094	0.177	0.249	0.281	0.28
	2	0.002	0.009	0.037	0.114	0.263	0.448	0.564

**Table 6:** Item category reference profile (ICRP) for distal items The ICRP calculates the probability of the patient for each of the latent ranks for each item of FMA-UE to be scored 0, 1, or 2 points on the prime score FMA-UE: upper extremity motor section of the Fugl-Meyer Assessment

Distal item	Score	Rank 1	Rank 2	Rank 3	Rank 4	Rank 5	Rank 6	Rank 7
Wrist stability, elbow at 90°	0	0.834	0.653	0.355	0.145	0.055	0.017	0.005
	1	0.121	0.219	0.337	0.33	0.205	0.078	0.025
	2	0.046	0.128	0.308	0.525	0.741	0.905	0.971
Wrist flexion/extension, elbow at 90°	0	0.813	0.621	0.319	0.112	0.031	0.006	0.001
	1	0.168	0.318	0.519	0.577	0.458	0.259	0.13
	2	0.02	0.061	0.163	0.311	0.511	0.735	0.869
Wrist stability, elbow at 0°	0	0.91	0.77	0.504	0.263	0.113	0.034	0.009
	1	0.079	0.188	0.356	0.414	0.312	0.156	0.07
	2	0.011	0.041	0.139	0.323	0.575	0.809	0.921
Wrist flexion/extension, elbow at 0°	0	0.889	0.745	0.472	0.227	0.087	0.024	0.006
	1	0.105	0.235	0.455	0.591	0.54	0.359	0.225
	2	0.005	0.021	0.074	0.182	0.372	0.616	0.769
Wrist circumduction	0	0.87	0.722	0.467	0.253	0.12	0.045	0.018
	1	0.129	0.274	0.513	0.675	0.69	0.598	0.513
	2	0.001	0.004	0.02	0.072	0.19	0.357	0.469
Finger mass flexion	0	0.373	0.268	0.12	0.033	0.006	0.001	0
	1	0.508	0.514	0.462	0.342	0.2	0.088	0.039
	2	0.119	0.219	0.418	0.625	0.794	0.912	0.961
Finger mass extension	0	0.616	0.446	0.201	0.053	0.009	0.001	0
	1	0.343	0.448	0.533	0.461	0.279	0.121	0.06
	2	0.041	0.106	0.267	0.486	0.712	0.878	0.94
Hook grasp	0	0.877	0.793	0.63	0.449	0.281	0.138	0.063
	1	0.091	0.136	0.197	0.215	0.168	0.093	0.052
	2	0.032	0.071	0.174	0.336	0.551	0.768	0.885
Abduct thumb	0	0.882	0.79	0.601	0.379	0.195	0.079	0.031
	1	0.105	0.176	0.303	0.409	0.405	0.302	0.225
	2	0.013	0.034	0.096	0.212	0.4	0.619	0.744
Oppose thumb and index finger pads	0	0.895	0.769	0.532	0.313	0.165	0.067	0.024
	1	0.09	0.183	0.328	0.397	0.331	0.19	0.1
	2	0.015	0.049	0.14	0.289	0.505	0.744	0.876
Cylindrical grasp	0	0.858	0.717	0.458	0.23	0.098	0.035	0.013
	1	0.113	0.207	0.349	0.409	0.334	0.189	0.098
	2	0.029	0.076	0.193	0.362	0.568	0.776	0.889
Spherical grasp	0	0.812	0.647	0.378	0.168	0.064	0.021	0.007
	1	0.139	0.229	0.343	0.366	0.266	0.125	0.049
	2	0.049	0.124	0.279	0.466	0.67	0.854	0.943

## Discussion

The results of the PCC and CFA indicated that the shoulder-elbow-forearm and coordination items, excluding the two reflex items, capture the ability of the proximal joints. In contrast, the wrist and finger items capture the ability of the distal joints, confirming the one-dimensionality of both areas. Additionally, a seven-layer structure was observed in both the proximal and distal items, with estimated TRP values tending to increase almost evenly across these layers. This finding supports the appropriateness of using seven layers when stratifying the severity of upper extremity motor paresis. While the previous study classified severity into three levels, mild, moderate, and severe, our results allowed for a seven-level stratification for both proximal and distal items, enabling a more detailed stratification of upper limb motor paralysis severity (Appendices) [7].

Furthermore, differences were observed in the order of items indicated by IRP compared to the item difficulty order presented by the previous study for both proximal and distal items (Table 4) [7]. Possible reasons for these differences may include variations in the number of excluded items. Nonetheless, we believe that our rank-based interpretation using LRT, in combination with the previous study, allows for new interpretations of the FMA-UE [7].

Through this study, it has become possible to interpret the degree of proximal and distal paralysis in stroke patients not only through the total FMA-UE score but also through subitems. Based on our findings, we propose a new recovery index. The seven ranks provide a new index distinct from previous studies. Appendices include examples of patient types represented by each rank for both proximal and distal items, with the scores of corresponding subitems emphasized. The identified seven proximal and distal ranks are expected to benefit future studies on stroke rehabilitation interventions by clarifying which types of stroke patients are likely to benefit most. For instance, the installed robots could use our findings to plan optimized training programs for each patient automatically.

Our findings have several limitations. We did not exclude subjects with biases related to lesion location, cognitive impairment, or the loss of somatosensory function. Additionally, since we were unable to follow up with participants longitudinally, we could not observe the actual recovery process of upper limb motor paralysis. However, as our study investigated FMA-UE in a large-scale, multicenter sample of stroke patients, it may reflect the clinical recovery index.

## Conclusions

This study found that the FMA-UE has seven latent ranks. Our findings may pave the way for future research that highlights the objective and clinical characteristics of stroke patients. The ranked results of the FMA-UE could serve as a valuable tool for interpreting the motor paralysis of specific patients, potentially promoting a more evidence-based approach to rehabilitation.

## Appendices

Interpretation of the type of patients represented by each of the ranks for proximal and distal items

Proximal Items

1. Rank 1 had a low score for all items and was considered to represent many patients with difficulty in voluntary movement and severe paralysis.

2. In rank 2, although an increase was observed in the item reference profile (IRP) for each item compared to rank 1, no significant increase in scores was observed in the item category reference profile (ICRP), and many participants had scores of 0 and 1 for all items. Therefore, rank 2 was dominated by patients who showed a slight appearance of voluntary movement.

3. Rank 3 patients showed an increase in the elbow flexion item of flexor synergy in the IRP, and the probability increased by two points in the ICRP. Therefore, rank 3 was considered to have more patients capable of elbow flexion of flexor synergy.

4. Compared to patients with rank 3, patients with rank 4 showed an increase in the IRP for the three items of extensor synergy, scapular elevation item of flexor synergy, and hand-to-lumbar spine item of combined movement and an increase of two points in the ICRP. Therefore, rank 4 is considered to represent patients who are more capable of extensor synergy, scapular elevation of flexor synergy, and hand-to-lumbar spine item of combined movement.

5. Compared to patients with rank 4, patients with rank 5 showed an increase in the IRP in items of shoulder abduction; scapular retraction; forearm supination; shoulder external rotation of the flexor synergy; forearm pronation/supination, elbow at 90°; and shoulder flexion to 90° of the combined movement and an increase of two points in the ICRP. Therefore, rank 5 was considered to represent patients more capable of flexor synergy; forearm pronation/supination, elbow at 90°; and shoulder flexion to 90° of combined movement.

6. Compared to patients with rank 5, patients with rank 6 showed an increase in the IRP in the items of shoulder abduction from 0° to 90° and forearm pronation/supination, elbow at 0°, of movement out of synergy and an increase of two points in the ICRP. Therefore, rank 6 is considered to represent patients who are more capable of shoulder abduction from 0° to 90° and forearm pronation/supination, elbow at 0°, of movement out of synergy.

7. Compared to patients with rank 6, patients with rank 7 showed an increase in the IRP in the coordination/speed and the item of shoulder flexion 90°-180° of the movement out of synergy and an increase of two points in the ICRP. The IRP score for the normal reflex was 0.970 points even at rank 7, which is a high-difficulty item, and it was difficult to obtain two points. Therefore, patients of rank 7 can be considered to perform items other than normal reflexes.

Distal Items

1. Rank 1 had a low score for all items, and many patients had difficulty with voluntary movements and were considered to have severe paralysis.

2. Compared to patients with rank 1, patients with rank 2 showed an increase in the IRP during finger mass flexion. Nevertheless, no significant increase in ICRP scores was observed, and many participants scored 0 or 1 for all items. Therefore, rank 2 was considered to represent patients capable of only slight finger mass flexion.

3. Compared to patients with rank 3, patients with rank 2 showed an increase in the IRP for multiple items. However, there was no marked increase in the ICRP, and many patients scored 0 or 1 point for all items. Therefore, rank 3 was considered to represent the participants who showed a slight appearance of voluntary movement in multiple items.

4. Compared to patients with rank 3, patients with rank 4 showed an increase in IRP in finger mass flexion and an increase of two points in ICRP. Therefore, rank 4 was considered to represent the participants capable of finger mass flexion.

5. Compared to patients with rank 4, patients with rank 5 showed an increase in the IRP for finger mass extension; wrist stability, elbow at 90°; and spherical grasp and an increase of two points in the ICRP. Therefore, rank 5 was considered to represent the participants capable of finger mass extension; wrist stability, elbow at 90°; and spherical grasp.

6. Compared to patients with rank 5, patients with rank 6 showed an increase in the IRP in the items of wrist flexion/extension, elbow at 90°; wrist stability, elbow at 0°; opposing thumb and index finger pads; cylindrical grasp; and hook grasp and an increase of two points in the ICRP. Therefore, rank 6 was considered to represent patients who were capable of flexion/extension, elbow at 90°; wrist stability, elbow at 0°; opposing thumb and index finger pads; cylindrical grasp; and hook grasp.

7. Compared to patients with rank 6, patients with rank 7 showed an increase in the IRP for wrist flexion/extension, elbow at 0°; thumb abduction; and wrist circumduction and an increase of two points in the ICRP. Therefore, rank 7 was considered to represent patients capable of performing all distal items.

Table 7 shows the FMA-UE and TRP values for proximal and distal items.

**Table 7:** Latent ranks in upper extremity motor section of the Fugl-Meyer Assessment (FMA-UE) and test reference profile (TRP) values for proximal and distal items

Latent ranks in the FMA-UE for proximal items	Number of participants (n)
Rank 1	113
Rank 2	22
Rank 3	50
Rank 4	63
Rank 5	60
Rank 6	48
Rank 7	153
Latent ranks in the FMA-UE for distal items	Number of participants (n)
Rank 1	122
Rank 2	27
Rank 3	39
Rank 4	62
Rank 5	57
Rank 6	42
Rank 7	160
TRP values for proximal items	Values
Rank 1	6.9
Rank 2	10.5
Rank 3	16.7
Rank 4	22.4
Rank 5	27.4
Rank 6	31.6
Rank 7	33.8
TRP values for distal items	Values
Rank 1	2.8
Rank 2	5.0
Rank 3	9.2
Rank 4	13.6
Rank 5	17.4
Rank 6	20.5
Rank 7	22.1
